# Supplementary material for: The state of iodine deficiency in Kyrgyzstan: Insights from studies of knowledge, attitudes and practices
Source: PLoS One. 2024 Nov 21;19(11):e0313869. doi: 10.1371/journal.pone.0313869 (PMC11581218; doi:10.1371/journal.pone.0313869)
Supplement: Attachment — Submitted filename: Response to reviewers.docx [file pone.0313869.s003.docx]

**Dear Dr Ramesh Athe and Reviewers,**

We sincerely thank you for the time and effort you have taken to review our manuscript, titled “The state of iodine deficiency in Kyrgyzstan: insights from studies of knowledge, attitudes and practices” (Manuscript ID: MS ID # PONE-D-24-31353). We greatly appreciate your thoughtful comments and suggestions, which have helped us to improve the quality of our work.

We have carefully considered all the feedback provided and have addressed each point in detail below. In response to your valuable suggestions, we have revised our manuscript, and we believe it has been substantially improved as a result.

**Reviewer #1:**

*Comment 1.1:* The figures in poor quality and hard to review.

*Response*: All figures are generated by a software and we cannot improve their quality except making bold the letters in names of axes.

*Comment 1.2:* Not clear whether the thyroid hormone levels and other health issues associated with UIC in these subjects?
*Response*: In our study, we did not check thyroid hormone levels and other health issues

*Comment 1.3:* Not sure whether the IDD in subclinical or clinical level?

*Response*: In the questionnaire, respondents were asked to answer, were they ever diagnosed any IDD.

*Comment 1.4:* Is there any relationship between low iodine and economic status?
*Response*: This issue is not highlighted in this paper since it is not the task of this paper. However, we did explore the association between UIC level and economic status (family income per capita) and highlighted it in our second manuscript submitted to this journal following this one.

**Reviewer #2:**

*Comment 2.1:* The introduction effectively sets the stage for the study. However, it could be improved by discussing the potential impact of cultural factors on iodine deficiency and how this study addresses those factors.

*Response*: We underlined the role of cultural factors in determining IDDs – lines 72-76.

*Comment 2.2:* The literature cited is comprehensive, but more recent studies on iodine deficiency and interventions could be included. This would help contextualize the findings within the broader global effort to combat iodine deficiency.

*Response*: Recent studies on iodine deficiency and interventions were mentioned in the review with focus on the findings within the broader global effort to combat iodine deficiency - lines 59-63.

*Comment 2.3:* The methodology is robust, but more details about the training of data collectors and the validation process of the questionnaire would enhance the reliability of the data. Additionally, the rationale for the chosen statistical methods should be explained in more detail.

*Response*: We improved description of the training of data collectors and the validation process of the questionnaire – lines 142-150.

*Comment 2.4:* The sampling method is well described, but it might be helpful to clarify how the pilot study influenced the final design of the questionnaire. Were there any significant changes based on the pilot study results?

*Response*: We clarify how the pilot study influenced the final design of the questionnaire and that it relusted in rephrasing and adjustment of its language – lines 141-145.

*Comment 2.5:* The multivariate regression model used to assess predictors of urinary iodine concentration (UIC) includes several variables (residence, knowledge, attitudes, practices, etc.). However, there is no mention of how multicollinearity between these predictors was assessed. Multicollinearity can inflate standard errors and make it difficult to determine the significance of individual predictors.

*Response*: We described how multicollinearity between these predictors was assessed in lines 171-174.

*Comment 2.6:* The study uses knowledge, attitude, and practice (KAP) scores as continuous variables in the regression analysis. While this is appropriate, the paper does not discuss whether these scores meet the assumptions of normality required for regression analysis. Additionally, the urinary iodine concentration (UIC) is also treated as a continuous variable, and its distribution is not examined.

*Response*: We described testing the assumptions of normality required for regression analysis in lines 184-186.

*Comment 2.7:* The presentation of the results is clear, but some sections could benefit from more detailed explanations. For example, in Table 3, it would be helpful to briefly explain the significance of the KAP scores and how they relate to each other in the text.

*Response*: We gave detailes of the significance of the KAP scores and how they relate to each other in lines 213-216.

*Comment 2.8:* Consider improving the readability of figures and tables. For example, Fig 4 (Relationships between KAP scores and UIC) could be more informative if the axes were labelled more clearly and the relationship explained in more detail within the figure caption.

*Response*: We have labelled axes S4 Fig clearer to indicate the relationship between KAP scores and UIC and added sentences – lines 301-306.

*Comment 2.9:* The discussion could be more concise. While it effectively interprets the findings, it tends to repeat points already made in the results section. Focus on the most critical findings and their implications.

*Response*: To avoid repeated points, we deleted some lines in the Results (lines 311-316) and in the Discussion sections (lines 330-335, 367-370, 435-437, 461-462) and reformulate some of them (lines 323-326, 370-374), added some sentences – lines 326-333, 462-464.

*Comment 2.10:* The discussion effectively highlights the gaps in knowledge and practices. However, it would be beneficial to compare the findings with other similar regions to give a more global perspective. Also, discussing the limitations in greater detail, particularly the cross-sectional design and potential biases, would strengthen the paper.

*Response*: Despite that in our manuscript, we have already compared our findings with other similar regions (lines 336-341, 343-345, 351-354, 363-366, 377-379, 408-416), we wrote additional sentences (lines 328-330, 417-420, 442-444).

*Comment 2.11:* Though the paper is structured properly, and the objectives are clear. However, the discussion section could benefit from more concise and focused arguments. Streamlining the findings and their implications will help the reader better understand the key takeaways.

*Response*: The study findings are streamlined in the introduction of the Discussion section and in the beginning each subsections (lines 323-324, 326-330, 356-359, 361-363, 398-407, 423-426). We reformulate some of them – lines 323-330. Their implications are give in lines 468-485.

*Comment 2.12:* The conclusions are sound but could be more actionable. It would be helpful to provide specific recommendations for policy changes or interventions based on the study’s findings. The conclusion should include more specific recommendations for public health interventions. Additionally, emphasizing the importance of ongoing monitoring and the role of healthcare providers in education could strengthen this section.

*Response*: We added two sentences with specific recommendations for policy changes or interventions providers in education based on the study’s findings stressing their improtance in lines 507-510.

*Comment 2.13:* While the language is generally clear, there are a few areas where sentences could be simplified for better readability. Proofreading for minor grammatical errors is recommended.

*Response*: For better readability and decrease redundancy in the text we deleted some sentences and added new ones – lines 310-316, 323-330.

*Comment 2.14:* Ensure that all references are up-to-date and relevant to the study. Some references could be replaced with more recent studies if available.

*Response*: We updated 11th, 25th, 28th, 29th, 30ties and 31st references and added anew, 26th and 33rd references.

We again thank the reviewers for their valuable feedback. We believe that the revisions have addressed all concerns raised and hope that the changes made are satisfactory. We look forward to your feedback and hope for a favorable outcome for the manuscript.

Sincerely,

**Kenesh Dzhusupov**

Department of Public Health, International Higher School of Medicine, Bishkek, Kyrgyzstan

Email: [k.dzhusupov@ism.edu.kg](mailto:k.dzhusupov@ism.edu.kg)
